# Inhibition of c-Jun NH_2_-terminal kinase or extracellular signal-regulated kinase improves lung injury

**DOI:** 10.1186/1465-9921-5-23

**Published:** 2004-11-27

**Authors:** Hui Su Lee, Hee Jae Kim, Chang Sook Moon, Young Hae Chong, Jihee Lee Kang

**Affiliations:** 1 Department of Physiology, Division of Cell Biology, Ewha Medical Research Institute, Ewha Womans University College of Medicine, 911-1 Mok-6-dong, Yangcheon-ku, Seoul 158-056, Korea; 2 Department of Microbiology, Division of Cell Biology, Ewha Medical Research Institute, Ewha Womans University College of Medicine, 911-1 Mok-6-dong, Yangcheon-ku, Seoul 158-056, Korea

**Keywords:** JNK, ERK, LPS, acute lung injury, NF-κB

## Abstract

**Background:**

Although in vitro studies have determined that the activation of mitogen-activated protein (MAP) kinases is crucial to the activation of transcription factors and regulation of the production of proinflammatory mediators, the roles of c-Jun NH_2_-terminal kinase (JNK) and extracellular signal-regulated kinase (ERK) in acute lung injury have not been elucidated.

**Methods:**

Saline or lipopolysaccharide (LPS, 6 mg/kg of body weight) was administered intratracheally with a 1-hour pretreatment with SP600125 (a JNK inhibitor; 30 mg/kg, IO), or PD98059 (an MEK/ERK inhibitor; 30 mg/kg, IO). Rats were sacrificed 4 hours after LPS treatment.

**Results:**

SP600125 or PD98059 inhibited LPS-induced phosphorylation of JNK and ERK, total protein and LDH activity in BAL fluid, and neutrophil influx into the lungs. In addition, these MAP kinase inhibitors substantially reduced LPS-induced production of inflammatory mediators, such as CINC, MMP-9, and nitric oxide. Inhibition of JNK correlated with suppression of NF-κB activation through downregulation of phosphorylation and degradation of IκB-α, while ERK inhibition only slightly influenced the NF-κB pathway.

**Conclusion:**

JNK and ERK play pivotal roles in LPS-induced acute lung injury. Therefore, inhibition of JNK or ERK activity has potential as an effective therapeutic strategy in interventions of inflammatory cascade-associated lung injury.

## Background

Lipopolysaccharide (LPS) causes acute lung injury associated with the activation of macrophages, an increase in alveolar-capillary permeability, neutrophil influx into the lungs, and parenchymal injury [1]. This pulmonary response contributes to the pathogenesis of various acute inflammatory respiratory diseases. Mitogen-activated protein (MAP) kinases are crucial in intracellular signal transduction, mediating cell responses to a variety of inflammatory stimuli, such as LPS, tumor necrosis factor (TNF) and interleukin (IL)-1. Recently, various *in vitro *studies have shown that pharmacological inhibitors of MAP kinases strongly affect the production of inflammatory mediators [2,3]. Through the use of specific inhibitors, the potential role of these kinases in inflammatory lung diseases is beginning to be studied. Treatment with p38 MAP Kinase inhibitors has been proposed as a selective intervention to reduce LPS-induced lung inflammation due to decreases in neutrophil recruitment to the air spaces [4,5]. However, the functions of c-Jun NH_2_-terminal kinase (JNK) and extracellular signal-regulated kinase (ERK) in LPS-induced lung injury remain unclear.

Cytokine-induced neutrophil chemoattractant (CINC) has been shown, in rodent models of lung injury, to play an important role in neutrophil migration into the lung [6]. Matrix metalloproteinases (MMPs), including MMP-9, allow activated neutrophils to permeate subsequent extracellular matrix (ECM) barriers after adhesion, and also for transendothelial cell migration, since these proteolytic enzymes digest most of the ECM components in the basement membranes and tissue stroma [7]. Another inflammatory mediator, nitric oxide (NO), has been linked to a number of physiologic processes, including leukocyte-dependent inflammatory processes and oxidant-mediated tissue injury [8,9]. Like CINC and MMP-9, overproduction of NO, which is dependent on the activity of inducible NO synthase, has been reported to contribute to endothelial or parenchymal injury, as well as to induce an increase in microvascular permeability, resulting in lung injury [10,11]. These inflammatory mediators are produced in response to LPS, TNF and IL-1 [6,11] and are regulated at the transcription level by nuclear factor-kappa B (NF-κB) [6,12].

NF-κB activation is regulated by phosphorylation of the inhibitor protein, IκB-α, which dissociates from NF-κB in the cytoplasm. The active NF-κB can then translocate to the nucleus, where it binds to the NF-κB motif of a gene promoter and functions as a transcriptional regulator. *In vivo *activation of NF-κB, but not other transcription factors, has also been demonstrated in alveolar macrophages from patients with acute respiratory distress syndrome (ARDS) [13]. Our previous study indicated that NF-κB activation is an important mechanism underlying both LPS-induced NO production, and also MMP-9 activity and resulting neutrophil recruitment [14]. Therefore, the activation of NF-κB binding to various gene promoter regions appears to be a key molecular event in the initiation of LPS-induced pulmonary disease.

Once activated, MAP kinases appear to be capable of further signal transduction through kinase phosphorylation, as well as modulating phosphorylation of transcription factors [15-17]. Activator protein (AP)-1, another transcription factor mediating acute inflammation, is activated through MAP kinase signaling cascades in response to various factors, such as LPS, cytokines, and various stresses and in turn regulates genes encoding inflammatory cytokines, such as TNF-α, IL-1, IL-6, and IL-8 [18]. Davis [19] reported that activated JNK is capable of binding the NH_2_-terminal activation domain of c-Jun, activating AP-1 by phosphorylating its component c-Jun. AP-1 can then translocate into the nucleus to promote transcription of downstream genes. However, action of MAP kinases on the upstream of NF-κB activation remains controversial [20-22]. Here, using a selective JNK inhibitor, SP600125, and the downstream MEK inhibitor of ERK, PD98059, we focused on the roles of JNK and ERK in LPS-induced acute lung injury and production of CINC, MMP-9, and NO. In addition, we investigated the regulatory effects of these MAP kinases on the NF-κB activation pathway during acute lung injury.

## Methods

### Experimental Animals

Specific pathogen-free male Sprague-Dawley rats (280–300 g) were purchased from Daehan Biolink Co. (Eumsung-Gun, Chungbuk, Korea). The Animal Care Committee of the Ewha Medical Research Institute approved the experimental protocol. The rats were cared for and handled according to the National Institute of Health (NIH) Guide for the Care and Use of Laboratory Animals.

### Experimental Protocols

Six groups of specific pathogen-free male Sprague-Dawley rats (280–300 g) were used: (1) controls received an intratracheal (IT) instillation of 0.5 ml of LPS-free saline (0.9 % NaCl); (2) an LPS-treated group received an IT instillation of 6 mg/kg body weight of LPS (*Escherichia coli *lipopolysaccharide, 055:B5, Sigma Chemical Co., St. Louis, MO) in 0.5 ml LPS-free saline; (3) an LPS-SP600125 group was injected with SP600125 (Calbiochem, La Jolla, CA) 1 hour before the IT instillation of 6 mg/kg body weight of LPS in 0.5 ml of LPS-free saline. (4) a saline-SP600125 group was injected with SP600125 1 hour before IT instillation of 0.5 ml of LPS-free saline (0.9 % NaCl); (5) an LPS-PD98059 group was injected with PD98059 (BIOMOL Research Laboratories, Plymouth, PA) 1 hour before IT instillation of 6 mg/kg body weight of LPS in 0.5 ml of LPS-free saline. (6) a saline-PD98059 group was injected with PD98059 1 hour before IT instillation of 0.5 ml of LPS-free saline (0.9 % NaCl). SP600125 or PD98059 was injected intraorally via a size 8 French feeding tube at a dose of 30 mg/kg body weight [5,23]. For IT instillation, rats were treated with enflurane anesthesia. The trachea was then exposed after a 1 cm midline cervical incision, and LPS or saline was injected intratracheally through a 24-gauge catheter. LPS or saline administration was immediately followed by 3 insufflations of 1 ml of air through the catheter and by rotating the animals to attempt to homogeneously distribute LPS or saline in the lungs. After a few minutes, the rats recovered from the anesthesia and were immediately placed in a chamber. Animals were sacrificed 4 hours after LPS treatment, and the following parameters were monitored: (1) phosphorylation of JNK, ERK, and p38 MAP kinase in lung tissue; (2) cell differential count, and measurement of protein content and lactate dehydrogenase (LDH) activity in bronchoalveolar lavage (BAL) fluid; (3) cytokine-induced neutrophil chemoattractant (CINC) expression, matrix metalloproteinase (MMP)-9 activity or expression and nitrite production in lung tissue, BAL fluid or the supernatants of alveolar macrophage cultures; (4) DNA binding activity of nuclear factor-kappa B (NF-κB) in lung tissue and alveolar macrophages; (5) serine phosphorylation and degradation of IκB-α in lung tissue. In addition, phosphorylation of JNK and ERK was also determined at 2, 4, 14 or 24 hours after LPS treatment to determine the kinetics of the kinase activation in lung tissue.

### Isolation of BAL cells, Lung Tissue, and Cell Counts

Four hours after LPS treatment, the rats were sacrificed, and BAL was then performed through a tracheal cannula with aliquots of 8 ml each using ice-cold Ca^2+^/Mg^2+^-free phosphate-buffered medium (145 mM NaCl, 5 mM KCl, 1.9 mM NaH_2_PO_4_, 9.35 mM Na_2_HPO_4_, and 5.5 mM dextrose; pH 7.4) for a total of 80 ml for each rat. The bronchoalveolar lavagate was centrifuged at 500 × *g *for 5 min at 4°C and cell pellets washed and resuspended in phosphate-buffered medium. Cell counts and differentials were determined using an electronic coulter counter with a cell sizing analyzer (Coulter Model ZBI with a channelizer 256; Coulter Electronics, Bedfordshire, UK), as described by Lane and Mehta [24]. Red blood cells, lymphocytes, neutrophils, and alveolar macrophages were distinguished by their characteristic cell volumes [25]. The recovered cells were 98% viable, as determined by trypan blue dye exclusion. Following lavage, lung tissue was removed, immediately frozen in liquid nitrogen, and stored at -70°C.

### Measurement of Total Protein and lactate dehydrogenase (LDH) Activity

To assess the permeability of the bronchoalveolar-capillary barrier, total protein was measured according to the method of Hartree [26], using bovine serum albumin as the standard. Total protein and LDH activity were measured in the first aliquot of the acellular BAL fluid. LDH activity, a cytosolic enzyme used as a marker for cytotoxicity, was measured at 490 nm using an LDH determination kit according to the manufacturer's instructions (Roche Molecular Biochemicals, Mannheim, Germany). LDH activity was expressed as U/L, using an LDH standard.

### Western Blot Analysis

Lung tissue homogenate samples (55 μg or 100 μg protein/lane for JNK, ERK, p38 MAP kinase, IκB-α and CINC) or aliquots of acellular BAL fluid (70 μl/lane for CINC and MMP-9) were separated on a 10% or 20% SDS-polyacrylamide gel. Separated proteins were electrophoretically transferred onto nitrocellulose paper and blocked for 1 hour at room temperature with Tris-buffered SAL containing 3% BSA. The membranes were then incubated with an anti-rabbit phospho-JNK/JNK antibody, anti-rabbit phospho-ERK/ERK, anti-rabbit phospho-p38 MAP kinase/p38 MAP kinase, antiserum against rat CINC, anti-human MMP-9 monoclonal antibody or anti-rabbit phospho-IκBα (Ser32)/IκBα at room temperature for 1 hour. Antibody labeling of protein bands was detected with enhanced chemiluminescence (ECL) reagents according to the supplier's protocol.

### Zymographic Analysis of MMP-9

The gelatinolytic activities in BAL fluid, or the supernatants of alveolar macrophage cultures, were determined using zymography with gelatin copolymerized with acrylamide in the gel according to previously published methods [14]. To obtain the supernatants of alveolar macrophage cultures, lavage cells were resuspended in RPMI-1640 medium (Mediatech, Washington, DC), containing 2 mM glutamine, 100 units/ml mycostatin without fetal bovine serum (FBS). Aliquots of 1 ml, containing 10^6 ^alveolar macrophages, were added to 24-well plates (Costar, Cambridge, MA) and incubated at 37°C in a humidified atmosphere of 5% CO_2 _for 2 hours. The nonadherent cells were then removed, and adherent cells were counted and further incubated in 1 ml RPMI medium. After a 24 hour incubation, the supernatant was collected and filtered.

Aliquots of BAL fluid and the culture supernatants, normalized for equal volume (8 μl) or amount of protein (8 μg), were electrophoresed on a 10% SDS-PAGE gel with 0.1% gelatin as a substrate without boiling under non-reducing conditions. After removing SDS with 2.5% Triton X-100 for 2 hours, gels were incubated for 20 hours at 37°C in 50 mM Tris-Cl (pH 7.4) containing 10 mM CaCl_2 _and 0.02% NaN_3_. The gels were then stained for 1 hour in 7.5% acetic acid/10% propanol-2 containing 0.5% Coomassie Brilliant Blue G250 and destained in same solution without dye. Positions of gelatinolytic activity are unstained on a darkly stained background. The clear bands on the zymograms were photographed on the negative (Polaroid's 665 film) and the signals were quantified by densitometric scanning using an UltroScan XL laser densitometer (LKB, Model 2222-020) to determine the intensity of MMP-9 activity as arbitrary densitometric units. To confirm MMP-9 activity, aliquots of BAL fluid were analyzed by Western blotting with anti-human MMP-9 monoclonal antibody, which was raised against MMP-9 secreted by human HT1080 fibrosarcoma cells [27] and cross-reacts with rat MMP-9 [28].

### Nitrite Assay in BAL fluid and Alveolar Macrophage Culture

NO levels in the first aliquot of the acellular BAL fluid, and the supernatants of alveolar macrophage cultures, were measured using a nitrite assay. Direct measurement of NO is difficult due to the very short half-life [29]. However, the stable oxidation end product of NO production, nitrite, can be readily measured in biological fluids and has been used *in vitro *and *in vivo *as an indicator of NO production [30]. Briefly, lavage cells were resuspended in RPMI-1640 medium (Mediatech, Washington, DC), containing 2 mM glutamine, 100 units/ml mycostatin, and 10% FBS. Aliquots of 1 ml, containing 10^6 ^alveolar macrophages were added to 24-well plates (Costar, Cambridge, MA) and incubated at 37°C in a humidified atmosphere of 5% CO_2 _for 2 hours. The non-adherent cells were then removed by vigorous washing with two 1 ml of RPMI medium. After incubating the cells for 24 hours, the supernatant was collected and filtered.

Nitrite was assayed after adding 100 μl Greiss reagent (1% sulfanilamide and 0.1% naphthylethylenediamide in 5% phosphoric acid) to 50 μl samples of BAL fluid and cell culture. Optical density at 550 nm (OD_550_) was measured using a microplate reader. Nitrite concentrations were calculated by comparison with OD_550 _of standard solutions of sodium nitrite prepared in cell culture medium. Data were presented as μM of nitrite.

### Nuclear Extracts

Nuclear extracts were prepared by a modified method of Sun *et al*. [31]. Lavage cells were resuspended in Dulbecco's modified Eagle's medium (DMEM; Mediatech, Washington, DC), supplemented with 5% FBS (HyClone, Logan, UT), 2 mM glutamine, and 1,000 units/ml penicillin-streptomycin. DMEM medium (5 ml), containing 5 × 10^6 ^alveolar macrophages, was added to 6-well plates and incubated at 37°C, in a humidified atmosphere of 5% CO_2 _for 2 hours. The nonadherent cells were then removed with two 1 ml aliquots of DMEM. At the end of the incubation, adherent cells (> 95% alveolar macrophages) were harvested and then resuspended in hypotonic buffer A (100 mM HEPES, pH 7.9, 10 mM KCl, 0.1 M ethylenediaminetetraacetic acid [EDTA], 0.5 mM dithiothreitol [DTT], 1% Nonidet P-40, and 0.5 mM phenylmethylsulfonyl fluoride [PMSF]) for 10 min on ice, then vortexed for 10 s. Nuclei were pelleted by centrifugation at 12,000 rpm for 30 s. Nuclear extracts were also prepared from lung tissue by the modified method of Deryckere and Gannon [32]. Aliquots of frozen tissue were mixed with liquid nitrogen and ground to powder using a mortar and pestle. The ground tissue was placed in a Dounce tissue homogenizer (Kontes Co., Vineland, NJ) in the presence of 4 ml of buffer A to lyse the cells. The supernatant containing intact nuclei was incubated on ice for 5 min, and centrifuged for 10 min at 5,000 rpm. Nuclear pellets obtained from alveolar macrophages or lung tissue were resuspended in buffer C (20 mM HEPES, pH 7.9, 20% glycerol, 0.42 M NaCl, 1 mM EDTA, and 0.5 mM PMSF) for 30 min on ice. The supernatants containing nuclear proteins were collected by centrifugation at 10,000 rpm for 2 min, and stored at -70°C.

### Electrophoretic Mobility Shift Assay (EMSA)

Binding reaction mixtures (10 μl), containing 5 μg (4 μl) nuclear extract protein, 2 μg poly (dI-dC)•poly (dI-dC) (Sigma Co., St. Louis. MO), and 40,000 cpm ^32^P-labeled probe in binding buffer (4 mM HEPES, pH 7.9, 1 mM MgCl_2_, 0.5 mM DTT, 2% glycerol, and 20 mM NaCl), were incubated for 30 min at room temperature. The protein-DNA complexes were separated on 5% non-denaturing polyacrylamide gels in 1 × TBE buffer, and autoradiographed. Autoradiographic signals for activated NF-κB were quantitated by densitometric scanning using an UltroScan XL laser densitometer (LKB, Model 2222-020, Bromma, Sweden) to determine the intensity of each band.

The oligonucleotide used as a probe for EMSA was a double-stranded DNA fragment, containing the NF-κB consensus sequence (5'-CCTGTGCTCCGGGAATTTCCCTGGCC-3'), labeled with [α-^32^P]-dATP (Amersham, Buckinghamshire, UK), using DNA polymerase Klenow fragment (Life Technologies, Gaithersburg, MD). Cold competition was performed by adding 100 ng unlabeled double-stranded probe to the reaction mixture.

### Statistical Analysis

Values were expressed as means ± standard errors. Data were compared among the groups by one-way ANOVA followed by a Tukey's *post hoc *test. A P value of < 0.05 was considered to be statistically significant.

## Results

### Phosphorylation of JNK and ERK in Lung Tissue

**Figure 1:**
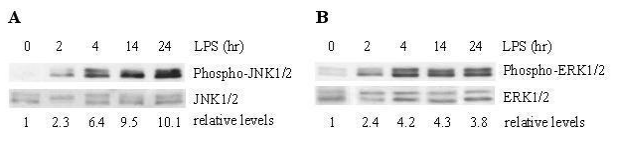
Time course of phosphorylation of JNK (A) and ERK (B), in lung tissue from rats treated with saline (0 time) or LPS (2–24 h). Western blots with anti-phospho-JNK/JNK antibody or phospho-ERK/ERK antibody were employed in order to monitor JNK or ERK phosphorylation. Relative values for levels of phosphorylated JNK1/2 or ERK1/2 normalized to JNK1/2 or ERK1/2 are indicated below the gel. Results are representative results from 5 rats in each group.

**Figure 2:**
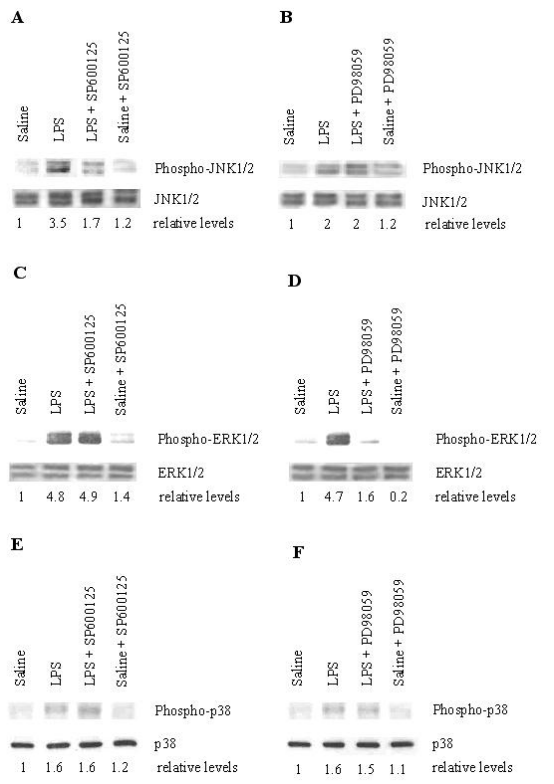
Phosphorylation of JNK (A, B), ERK (C, D)and p38 MAP kinase (E, F) in lung tissue 4 hours after saline or LPS treatment. The groups represent rats treated as follows: Saline, saline (IT); LPS, LPS (IT); LPS-SP600125, LPS (IT) and a pretreatment with SP600125 (IO), Saline-SP600125, saline (IT) and a pretreatment with SP600125 (IO);LPS-PD98059, LPS (IT) and a pretreatment with PD98059 (IO), Saline-PD98059, saline (IT) and a pretreatment with PD98059 (IO). Western blots with anti-phospho-JNK/JNK antibody, phospho-ERK/ERK antibody or phospho-p38 MAP kinase/p38 MAP kinase were employed in order to monitor JNK, ERK or p38 MAP kinase phosphorylation. Relative values for levels of phosphorylated JNK1/2, ERK1/2 or p38 MAP kinse normalized to JNK1/2, ERK1/2 or p38 MAP kinase are indicated below the gel. Results are representative results from 5 rats in each group.

To determine JNK and ERK activation in the lung tissue from LPS treated animals, Western blot analysis with a phospho-specific JNK antibody or ERK antibody was employed. Figures 1A and 1B showed time courses of LPS-induced phosphorylation, or activation, of JNK1/2 and ERK1/2. Phosphorylation of these MAP kinases substantially increased beginning 4 hours after LPS treatment, and progressively further increased (JNK activation) or were maintained (ERK activation) for up to 24 hours after LPS treatment. SP600125 pretreatment partially inhibited LPS-induced phosphorylation of JNK1/2 in lung tissue at 4 hours after LPS treatment (Figure 2A), but this inhibitor had little effect on the activation of ERK1/2 (2C) and p38 MAP kinse (2E). PD98059 pretreatment specifically inhibited the activation of ERK1/2 (Figure 2D), but neither the activation of JNK1/2 (2B) nor p38 MAP kinase (2F). Both JNK and ERK activation were barely detectable in the animals treated with saline or saline-kinase inhibitors.

### Total Protein and LDH Activity in BAL Fluid and Neutrophil Influx into Lungs

**Figure 3:**
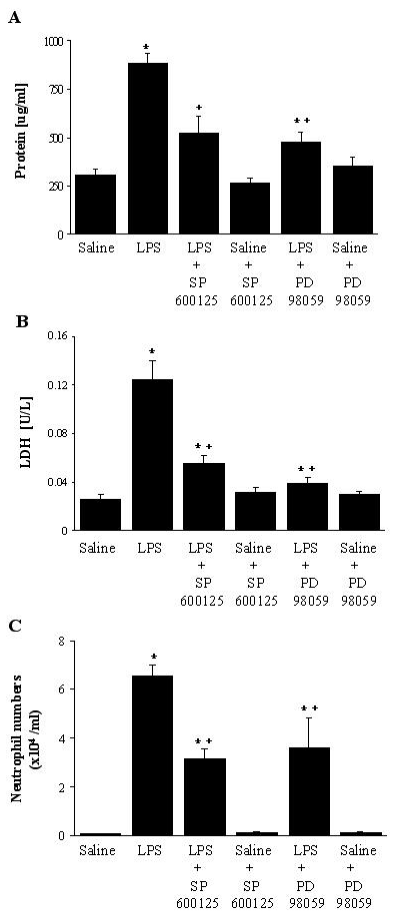
Levels of total protein (A), activity of LDH (B) and neutrophil numbers (C) in bronchoalveolar lavage fluid. The groups represent rats treated as follows: Saline, saline (IT); LPS, LPS (IT); LPS-SP600125, LPS (IT) and a pretreatment with SP600125 (IO), Saline-SP600125, saline (IT) and a pretreatment with SP600125 (IO);LPS-PD98059, LPS (IT) and a pretreatment with PD98059 (IO), Saline-PD98059, saline (IT) and a pretreatment with PD98059 (IO). Animals were sacrificed 4 hours after LPS treatment. Values represent means ± SEM of results from 5 rats in each group. * Significant differences between saline, p < 0.05, and + significant difference compared with LPS group, p < 0.05.

BAL protein contents (Figure 3A) and LDH activity (Figure 3B) in LPS-treated animals were significantly increased (p < 0.05). BAL protein increased 2.9-fold, and LDH activity increased 4.7-fold. This indicates that IT LPS treatment of rats induced acute lung injury. However, SP600125 or PD98059 pretreatment significantly inhibited LPS-induced changes in protein contents, by 63 and 74%, respectively, and BAL LDH activity by 71 and 86%, respectively (P < 0.05). There were no significant differences in these parameters between saline-SP600125, saline-PD98059, and saline control animals (p < 0.05).

BAL cells were differentially analyzed, in order to evaluate the effects of these kinase inhibitors on LPS-induced neutrophil influx. As shown in Figure 3C, neutrophil counts of the total lung lavage cells in LPS-treated animals significantly increased by a factor of 26, compared to values in saline-treated animals, indicating a significant increase in neutrophil influx into the alveolar spaces (p < 0.05). SP600125 or PD98059 significantly suppressed BAL neutrophil counts by 53 or 46 %, respectively (*vs *LPS animals, p < 0.05). The BAL neutrophil counts in saline-kinase inhibitor animals were not significantly different from those of the saline control animals (p < 0.05).

### CINC, MMP-9 and NO Production in Lungs or Alveolar Macrophages

**Figure 4:**
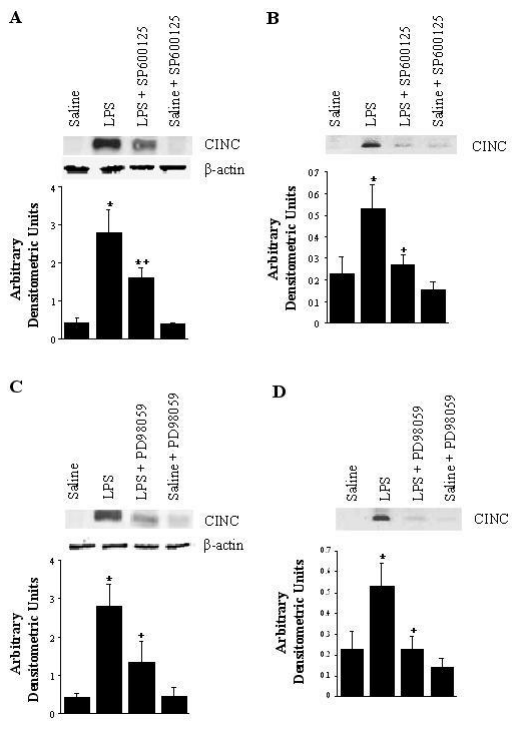
CINC expression in lung tissue (A, C) and bronchoalveolar lavage fluid (B, D). The groups represent rats treated as follows: The groups represent rats treated as follows: Saline, saline (IT); LPS, LPS (IT); LPS-SP600125, LPS (IT) and a pretreatment with SP600125(IO), Saline-SP600125, saline (IT) and a pretreatment with SP600125(IO); LPS-PD98059, LPS (IT) and a pretreatment with PD98059 (IO), Saline-PD98059, saline (IT) and a pretreatment with PD98059 (IO). Animals were sacrificed 4 hours after LPS treatment. Western blots with anti-CINC antibodies were performed on the samples of lung tissue and BAL fluid. Densitometry of CINC bands is expressed in arbitrary densitometric units. Values are represented as means ± SEM of results from 5 rats in each group. * Significant differences between saline, p < 0.05, and + significant difference compared with LPS group, p < 0.05.

CINC, MMP-9 and NO were chosen in our experiments as representative inflammatory mediators, because of their important roles in neutrophil influx and lung damage, and also because their gene regulation is dependent on NF-κB. Figure 4 illustrates representative Western blots of lung tissue and BAL fluid for CINC. CINC protein expression was undetectable in the samples of saline control animals, but was markedly increased by LPS treatment for 4 hours. By densitometric analysis, CINC protein in lung tissue (Figure 4A and 4C*lane 2*) and BAL fluid (Figure 4B and 4D*lane 2*) from LPS animals was approximately 7- and 2.5-fold higher than in saline control animals, respectively. SP600125 or PD98059 significantly decreased the level of LPS-induced CINC expression, by 50 and 62%, respectively, in lung tissue (Figure 4A and 4C*lane 3*, p < 0.05) and, by 76 and 97%, respectively, in BAL fluid (Figure 4B and 4D*lane 3*, p < 0.05). These kinase inhibitors alone had little effect on CINC levels in the lung tissue and lavage fluid.

**Figure 5:**
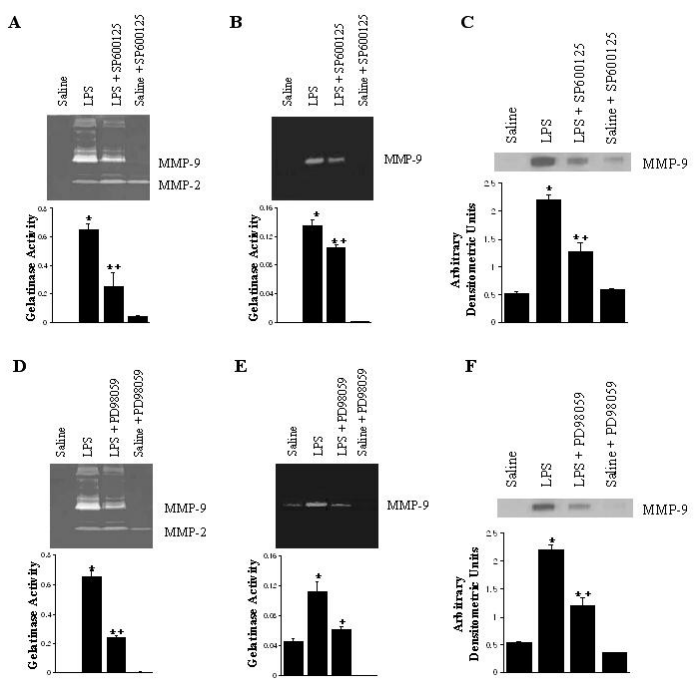
Gelatinolytic activities in bronchoalveolar lavage fluid (A, D) and the supernatants of alveolar macrophages in culture (B, E). MMP-9 expression in bronchoalveolar lavage fluid (C, F). The groups represent rats treated as follows: Saline, saline (IT); LPS, LPS (IT); LPS-SP600125, LPS (IT) and a pretreatment with SP600125(IO), Saline-SP600125, saline (IT) and a pretreatment with SP600125 (IO); LPS-PD98059, LPS (IT) and a pretreatment with PD98059 (IO), Saline-PD98059, saline (IT) and a pretreatment with PD98059 (IO). Animals were sacrificed 4 hours after LPS treatment. Alveolar macrophages (10^6^/m1 of RPMI medium) were incubated for 24 hours. BAL fluid and culture supernatants were analyzed by sensitive zymography, followed by scanning densitometry. 92 kD and 66 kD gelatinolytic bands correspond to MMP-9 and MMP-2, respectively. Densitometry of 92 kD bands is expressed in arbitrary densitometric units. Western blots of BAL fluid with anti-MMP-9 antibodywere employed to monitor MMP-9. Values are represented as means ± SEM of results from 5 rats in each group. * Significant differences between saline, p < 0.05, and + significant difference compared with LPS group, p < 0.05.

BAL fluid (Figure 5A and 5D), and the supernatants from alveolar macrophage cultures (Figure 5B and 5E), were analyzed for evidence of MMP-9 activity, using gelatin zymography. The BAL fluid from the saline control animals showed undetectable gelatinolytic bands. LPS treatment induced a distinct increase in the amount of gelatinolytic activity and the most prominent band was found to be a 92 kD species in the BAL fluid, corresponding to a molecular weight identical to MMP-9 [25,26]. This was confirmed to be MMP-9 by Western blot analysis with the antiMMP-9 monoclonal antibody (Figure 5C and 5F*lane 2*). In the supernatants from alveolar macrophage cultures of saline control animals, MMP-9 activity was also barely detectable, but was also markedly increased in the sample from LPS animals. SP600125 pretreatment significantly inhibited LPS-induced MMP-9 activity by 54% in BAL fluid, and by 30% in the supernatants from alveolar macrophage cultures (Figure 5A and 5B*lane 3*, p < 0.05). Similarly, PD98059 pretreatment significantly inhibited LPS-induced MMP-9 activity by approximately 67% in BAL fluid and the supernatants from alveolar macrophage cultures (Figure 5D and 5E*lane 3*, p < 0.05). MMP-9 activity was completely undetectable in the samples from saline-kinase inhibitor animals. The inhibitory effect of these kinase inhibitors on MMP-9 expression in BAL fluid was also observed (Figure 5C and 5F*lane 3*).

**Figure 6:**
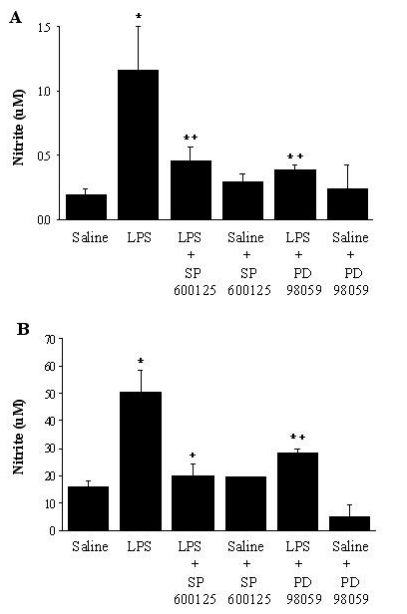
NO production in BAL fluid (A) and alveolar macrophages in culture (B). The groups represent rats treated as follows: Saline, saline (IT); LPS, LPS (IT); LPS-SP600125, LPS (IT) and a pretreatment with SP600125 (IO), Saline-SP600125, saline (IT) and a pretreatment with SP600125 (IO); LPS-PD98059, LPS (IT) and a pretreatment with PD98059 (IO), Saline-PD98059, saline (IT) and a pretreatment with PD98059 (IO). Animals were sacrificed 4 hours after LPS treatment. Alveolar macrophages (10^6^/m1 of RPMI medium) were incubated for 24 hours. BAL fluid and culture supernatants were analyzed using nitrite assays. Values are represented as means ± SEM of results from 5 rats in each group. * Significant differences compared with saline, p < 0.05, and + significant difference compared with LPS group, p < 0.05.

NO levels in BAL fluid and alveolar macrophages in culture were determined by measurement of nitrite in their supernatants. Figure 6A illustrates that *in vivo *exposure to LPS for 4 hours resulted in a 6.2-fold increase in NO level in BAL fluid, compared with the control animals. This increase was significantly inhibited by SP600125 or PD98059 (69% and 81% inhibition, respectively, p < 0.05, *vs *LPS animals). In LPS treated animals, nitrite production from alveolar macrophages cultured for 24 hours was increased 3.2-fold (Figure 6B). SP600125 or PD 98059 significantly suppressed LPS-induced NO production by alveolar macrophages by 89 and 58%, respectively (p < 0.05). NO level was only slightly changed in the samples from saline-kinase inhibitor animals.

### NF-κB Activation in Lung Tissue and Alveolar Macrophages

**Figure 7:**
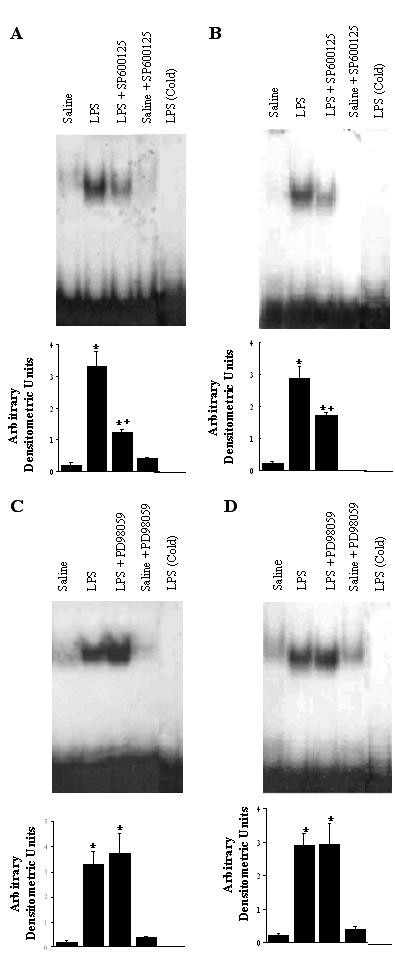
EMSA illustrating DNA-binding activity of NF-κB to the NF-κB motif in lung tissue (A, C), and alveolar macrophages (B, D). The groups represent rats treated as follows: Saline, saline (IT); LPS, LPS (IT); LPS-SP600125, LPS (IT) and a pretreatment with SP600125 (IO), Saline-SP600125, saline (IT) and a pretreatment with SP600125 (IO); LPS-PD98059, LPS (IT) and a pretreatment with PD98059 (IO), Saline-PD98059, saline (IT) and a pretreatment with PD98059 (IO). Animals were sacrificed 4 hours after LPS treatment. Nuclear extracts were prepared in lung tissue and alveolar macrophages (5 × 10^6 ^alveolar macrophages). Addition of 100 ng of unlabeled cold competitor to the LPS samples successfully competed for NF-κB binding, and eliminated the specific band. Densitometry of NF-κB bands on EMSA is expressed in arbitrary densitometric units. Values are represented as means ± SEM of results from 5 rats in each group. * Significant differences compared with saline, p < 0.05, and + significant difference compared with LPS group, p < 0.05.

Figure 7 shows NF-κB activation in lung tissue and alveolar macrophages, identified 4 hours after IT instillation of saline or LPS. In LPS-treated animals, the DNA-binding activities of NF-κB in lung tissue were markedly enhanced (Figure 7A and 7C*lane 2*). This enhancement was significantly depressed (67% inhibition, p < 0.05, *vs *LPS animals) by a 1-hour pretreatment with SP600125 (Figure 7A*lane 3*), whereas pretreatment with PD98059 did not inhibit LPS-induced activation of NF-κB (7C *lane 3*). Significant activation of NF-κB was also shown in alveolar macrophages from LPS-treated animals, compared to that seen in the saline control animals (Figure 7B and 7D*lane 2*, p < 0.05). SP600125 resulted in significant decreases (45%, p < 0.05) in LPS-induced NF-κB activation in alveolar macrophages (Figure 7B*lane 3*). However, PD98059 did not inhibit LPS-induced NF-κB activation in alveolar macrophages (Figure 7D*lane 3*). Saline or kinase inhibitors alone had little effect on NF-κB activation in lung tissue and alveolar macrophages.

The addition of the cold competitor eliminated the specific bands in the samples from LPS-treated animals, indicating that the band on the autoradiogram was specific for NF-κB binding (Figure 7A,7B,7C and 7D*lane 5*).

### Phosphorylation and Degradation of IκB-α in Lung Tissue

**Figure 8:**
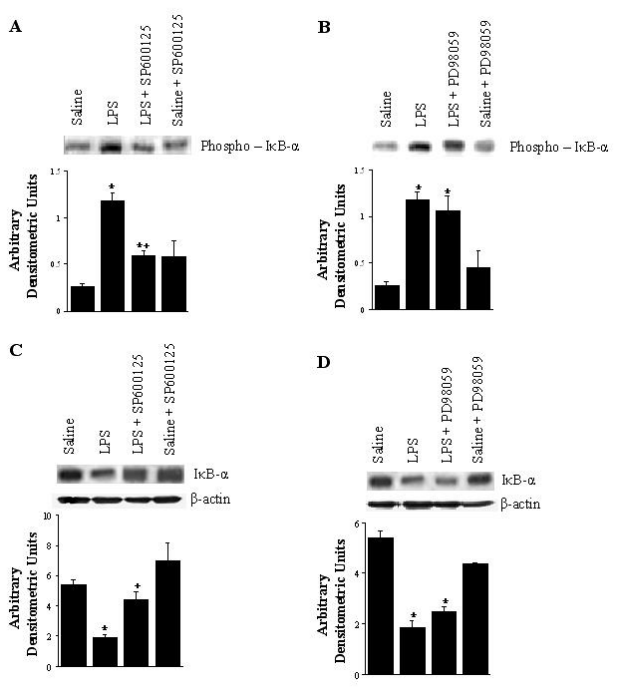
Phosphorylation (A, B) and degradation (C, D) of IκB-α in lung tissue. The groups represent rats treated as follows: Saline, saline (IT); LPS, LPS (IT); LPS-SP600125, LPS (IT) and a pretreatment with SP600125 (IO), Saline-SP600125, saline (IT) and a pretreatment with SP600125 (IO); LPS-PD98059, LPS (IT) and a pretreatment with PD98059 (IO), Saline-PD98059, saline (IT) and a pretreatment with PD98059 (IO). Animals were sacrificed 4 hours after LPS treatment. Western blots with anti-serine phospho-IκBα (Ser32)/IκBα antibody were employed to monitor phosphorylated IκB-α and IκB-α. Densitometry of phospho-IκB-α/IκB-α bands is expressed in arbitrary densitometric units. Values represent means ± SEM of results from 5 rats in each group. * Significant differences compared with saline, p < 0.05, and + significant difference compared with LPS group, p < 0.05.

In order to investigate a possible mechanism underlying the actions of these kinase inhibitors on LPS induction of pathways leading to NF-κB activation, serine phosphorylation and degradation of IκB-α, in lung tissue from LPS and LPS-kinase inhibitor animals were analyzed by Western blotting with anti-phospho-IκB-α (serine 32), and anti-IκB-α Ab. As shown in Figure 8, LPS treatment resulted in the induction of serine phosphorylation of IκB-α, and a substantial reduction in IκB-α protein content in lung tissue (8A and 8C *lane 2*), whereas these events were significantly inhibited by SP600125 (8A and 8C *lane 3*). PD98059, however, caused no significant changes in the LPS-induced phosphorylation and degradation of IκB-α (Figure 8B and 8D*lane 3*). These kinase inhibitors, alone, had little effect on the phosphorylation and degradation of IκB-α.

## Discussion

In the present study, we determined: (1) the *in vivo *relation between activation of JNK or ERK and LPS-induced acute lung injury; (2) the inhibition of JNK or ERK resulted in reductions of LPS-induced increases in lung injury parameters, such as total protein content and LDH activity in BAL fluid, neutrophil influx into the lungs, and proinflammatory gene products, such as CINC, MMP-9 and NO; and (3) the activation of JNK is involved in the LPS signaling pathway leading to NF-κB activation through phosphorylation of IκB-α and sequential degradation of IκB-α, whereas activation of ERK signaling is not associated with these NF-κB pathways.

JNK and ERK are known to play important roles as upstream regulators of the induced expression of inflammatory mediators in response to cytokines, stress, and cytoskeletal reorganization [19,33]. However, these results were exclusively obtained during *in vitro *experiments. Therefore, it is important to clarify the functions of these MAP kinases under pathological conditions. Furthermore, up to date, there have been no studies addressing the issues of the significance of JNK and ERK signaling in LPS-induced acute lung injury. Data from the present study clearly indicate that JNK and ERK were both activated after *in vivo *LPS exposure in models of acute lung injury. The degree of JNK and ERK activation from the baseline seems to be similar between these kinases, and to be generally higher than that of p38 MAP kinase (data not shown). The time course of JNK activation (progressive increase up to 24 hours after LPS treatment) appears to occur in parallel with those of the biochemical lung injury variables and neutrophil influx into the lungs after intratracheal treatment with LPS [14]. Consistent with our *in vivo *data, Ishii et al. [34] have reported that both JNK and ERK were simultaneously activated in the lung during ischemia and reperfusion. To explore a new therapeutic strategy for preventing the occurrence of LPS-induced lung inflammation and injury, we attempted to inhibit JNK or ERK activity by pretreatment with a selective JNK inhibitor, SP600125, or a specific inhibitor of MEK/ERK, PD98059, 1 hour before LPS treatment. Inhibition of either JNK or ERK activity resulted in reduction of LPS-induced increases in lung injury parameters, as well as neutrophil influx into the lungs, indicating that aggravating signals related to both JNK and ERK are associated with LPS-induced acute lung injury.

Since the increases in production or activity of CINC, MMP-9 and NO in BAL fluid were correlated with neutrophil influx and lung injury [6,8,9,11], the inhibitory effects of JNK or ERK on production of these inflammatory mediators are functionally important, and can thus be adopted as therapeutic interventions. Recently, it has been demonstrated that JNK inhibition by SP600125, in both ischemia and reperfusion periods, almost completely suppressed TNFα release into BAL fluid [34] and IL-1-induced MMP expression in synoviocytes and in joint arthritis [23]. These *in vivo *data with the specific JNK inhibitor are clearly consistent with an aggravating role of JNK in inflammation and tissue destruction. However, JNK1-/- mice have increased susceptibility to hyperoxia-induced lung injury [35]. Through *in vitro *studies of primary monocytes and macrophage cell lines, ERK has been linked to production of MMP-9, and to IL-8 release in response to LPS [36] or *Helicobacter pylori *[16]. Consistent with our data with ERK, Zhang et al. [37] reported that inhibition of ERK by PD98059 *in vivo *suppressed hyperoxia-induced cell death in lung tissues. ERK, however, also plays a protective role in myocardial ischemia/reperfusion injury [38], and in myoglobinuric acute renal injury [39]. Taken together, the primary role of JNK or ERK may play either a protective or injurious role under different experimental conditions.

The activation of NF-κB has been associated with lung injury in LPS- and silica-treated rats [6,40,41], and patients with ARDS [42] and asthma [43]. Data from our previous study indicate that *in vivo *activation of NF-κB in LPS-treated rats preceded the transcription of genes for proinflammatory mediators or lung inflammation and injury, i.e. the increases in neutrophil numbers, total protein, and LDH activity in BAL fluid [9]. These results suggest that NF-κB is an important intracellular target for the early detection and prevention of lung injury. Since the NF-κB element is believed to be the main regulator of CINC, MMP-9 and iNOS expression, and MAP kinase pathways have been demonstrated to contribute to the activation of NF-κB [15,44,45], we attempted to investigate the action of MAP kinases on the upstream of NF-κB signal transduction pathways in LPS-induced acute lung injury. Data from the present study indicate that inhibition of JNK suppresses LPS-induced increases in the DNA binding activity of NF-κB, through down-regulation of phosphorylation and degradation of IκB-α. Recently, Leonardi et al. [46] have reported that CIKS, a NF-κB essential modulator (NEMO)/IκB-α kinase (IKK)γβ-associated protein, connects to both the IKK and JNK signaling complexes, and activates an NF-κB-dependent reporter. Consequently, our and Leonardi et al.'s data support the possibility of JNK's role as an upstream activator of NF-κB, although the apparent complexity may reflect the fact that many diverse signals affect these two pathways. However, inhibition of ERK did not influence LPS induction of NF-κB activation, or the phosphorylation and degradation of IκB-α. The action of MAP kinases on the upstream of NF-κB pathways remains controversial in the context of in vitro experiments [20,47-51]. For instance, over-expression of either MEK1 or ERK1 resulted in a constitutive nuclear localization of NF-κB DNA binding activity [47]. Conversely, transfection with dominant negative MEK1 suggested that the ERK pathway does not regulate NF-κB DNA binding, stimulated by *Helicobacter pylori *[49]. Considering, by inference, a critical role of NF-κB in the LPS-induced inflammatory cascade, we speculate that ERK may exert effects on gene expression of these inflammatory mediators by modulating TATA-binding protein activation without affecting DNA binding activity [21]. Further study is absolutely necessary, however, to clarify the pathways of these MAP kinases associated with NF-κB, as well as other transcription factors, including AP-1 and cyclic adenosine 5'-monophosphate response element-binding protein (CREB).

## Conclusion

Data from this study suggest that JNK or ERK activation would play a detrimental role in LPS-induced acute lung injury. The inhibition of JNK or ERK should effectively block amplification of the LPS-induced catalytic cascade, through significant reductions in protein leakage, LDH release and neutrophil influx into the lung, and also in levels of CINC, MMP-9 and NO after LPS treatment. In addition, the inhibition of JNK activity, but not ERK activity, led to a decrease in NF-κB DNA binding activity through the suppression of phosphorylation and degradation of IκB-α. Based on these findings, we propose that inhibition of JNK or ERK may be an effective therapeutic strategy against the early stages of lung injury, via attenuation of the inflammatory cascade.

## Abbreviations

MAP; mitogen-activated protein, JNK; c-Jun NH_2_-terminal kinase, ERK; extracellular signal-regulated kinase, LPS; lipopolysaccharide, NF-κB; nuclear factor-kappa B, TNF; tumor necrosis factor, IL; interleukin, CINC; cytokine-induced neutrophil chemoattractant, MMP; matrix metalloproteinase, ECM; extracellular matrix NO; nitric oxide, ARDS; acute respiratory distress syndrome, IT; intratracheal, LDH; lactate dehydrogense, BAL; bronchoalveolar lavage, FBS; fetal bovine serum, DMEM; Dulbecco's modified Eagle's medium, EMSA; Electrophoretic Mobility Shift Assay, NEMO; NF-κB essential modulator (NEMO), IKK;IκB-α kinase, AP; activator protein, CREB; cyclic adenosine 5'-monophosphate response element-binding protein

## Authors' contributions

HSL, HJK and CSK carried out animal studies. HSL performed the statistical analysis. YHC participated in the zymographic analysis. JLK conceived of the study, participated in its design and coordination, and drafted and edited the manuscript. All authors read and approved the final manuscript.

## References

[B1] Steinberg KP, Milberg JA, Martin TR, Mnunder RJ, Cockrill BA, Hudson LD (1997). Evolution of bronchoalveolar cell populations in the adult respiratory distress syndrome. Am J Respir Crit Care Med.

[B2] van den Blink B, Juffermans NP, ten Hove T, Schultz MJ, van Deventer SJ, van der Poll T, Peppelenbosch MP (2001). p38 mitogen-activated protein kinase inhibition increases cytokine release by macrophages in vitro and during infection in vivo. J Immunol.

[B3] Scherle PA, Jones EA, Favata MF, Daulerio AJ, Covington MB, Nurnberg SA, Magolda RL, Trzaskos JM (1998). Inhibition of MAP kinase kinase prevents cytokine and prostaglandin E2 production in lipopolysaccharide-stimulated monocytes. J Immunol.

[B4] Nick JA, Young SK, Brown KK, Avdi NJ, Arndt PG, Suratt BT, Janes MS, Henson PM, Worthen GS (2000). Role of p38 mitogen-activated protein kinase in a murine model of pulmonary inflammation. J Immunol.

[B5] Underwood DC, Osborn RR, Bochnowicz S, Webb EF, Rieman DJ, Lee JC, Romanic AM, Adams JL, Hay DW, Griswold DE (2000). SB 239063, a p38 MAPK inhibitor, reduces neutrophilia, inflammatory cytokines, MMP-9, and fibrosis in lung. Am J Physiol Lung Cell Mol Physiol.

[B6] Blackwell TS, Blackwell TR, Holden EP, Christman BW, Christman JW (1996). In vivo antioxidant treatment suppresses nuclear factor-kappa B activation and neutrophilic lung inflammation. J Immunol.

[B7] Nagase H, Woessner JF (1999). Matrix metalloproteinases. J Biol Chem.

[B8] Kubes P (1995). Nitric oxide affects microvascular permeability in the intact and inflamed vasculature. Microcirculation.

[B9] Kang JL, Lee HW, Lee HS, Pack IS, Chong Y, Castranova V, Koh Y (2001). Genistein prevents nuclear factor-kappa B activation and acute lung injury induced by lipopolysaccharide. Am J Respir Crit Care Med.

[B10] Koh Y, Kang JL, Park W, Pack IS, Lee HS, Kim MJ, Lim CM (2001). Inhaled nitric oxide down-regulates intrapulmonary nitric oxide production in lipopolysaccharide-induced acute lung injury. Crit Care Med.

[B11] Kristof AS, Goldberg P, Laubach V, Hussain SN (1998). A Role of inducible nitric oxide synthase in endotoxin-induced acute lung injury. Am J Respir Crit Care Med.

[B12] Kumar A, Dhawan S, Mukhopadhyay A, Aggarwal BB (1999). Human immunodeficiency virus-1-tat induces matrix metalloproteinase-9 in monocytes through protein tyrosine phosphatase-mediated activation of nuclear transcription factor NF-kappaB. FEBS Lett.

[B13] Schwartz MD, Moore EE, Moore FA, Shenkar R, Moine P, Haenel JB, Abraham E (1996). Nuclear factor-kappa B is activated in alveolar macrophages from patients with acute respiratory distress syndrome. Crit Care Med.

[B14] Kang JL, Lee HW, Lee HS, Pack IS, Castranova V, Koh Y (2003). Time course for inhibition of lipopolysaccharide-induced lung injury by genistein: relationship to alteration in nuclear factor-kappaB activity and inflammatory agents. Crit Care Med.

[B15] Carter AB, Knudtson KL, Monick MM, Hunninghake GW (1999). The p38 mitogen-activated protein kinase is required for NF-κB-dependent gene expression. The role of TATA-binding protein (TBP). J Biol Chem.

[B16] Bhattacharyya A, Pathak S, Datta S, Chattopadhyay S, Basu J, Kundu M (2002). Mitogen-activated protein kinases and nuclear factor-kappaB regulate Helicobacter pylori-mediated interleukin-8 release from macrophages. Biochem J.

[B17] Jacobs AT, Ignarro LJ (2003). Nuclear factor-κB and mitogen-activated protein kinases mediate nitric oxide-enhanced transcriptional expression of interferon-β. J Biol Chem.

[B18] Shaulian E, Karin M (2002). AP-1 as a regulator of cell life and death. Nat Cell Biol.

[B19] Davis RJ (2000). Signal transduction by the JNK group of MAP kinases. Cell.

[B20] Kumar A, Middleton A, Chambers TC, Mehta KD (1998). Differential roles of extracellular signal-regulated kinase-1/2 and p38 (MAPK) in interleukin-1β-and tumor necrosis factor-α-induced low density lipoprotein receptor expression in HepG2 cells. J Biol Chem.

[B21] Carter AB, Hunninghake GW (2000). A constitutive active MEK → ERK pathway negatively regulates NF-kappa B-dependent gene expression by modulating TATA-binding protein phosphorylation. J Biol Chem.

[B22] Matthews JS, O'Neill LA (1999). Distinct roles for p42/p44 and p38 mitogen-activated protein kinases in the induction of IL-2 by IL-1. Cytokine.

[B23] Han Z, Boyle DL, Chang L, Bennett B, Karin M, Yang L, Manning AM, Firestein GS (2001). c-Jun N-terminal kinase is required for metalloproteinase expression and joint destruction in inflammatory arthritis. J Clin Invest.

[B24] Lane FC, Mehta JR (1990). In vitro human tumor sensitivity assay using cell counting and sizing. Am Biotechnol Lab.

[B25] Castranova VT, Jones MW, Barger A, Afshari, Frazer DJ, Jacobs RR, Wakelyn PJ (1990). Pulmonary responses of guinea pigs to consecutive exposures to cotton dust. Proceedings of the fourteenth Cotton Dust Research Conference: Memphis.

[B26] Hartree EF (1972). Determination of protein: a modification of the Lowry method that gives a linear photometric response. Anal Biochem.

[B27] Sato H, Takahisa T, Okada Y, Cao J, Shinagawa A, Yamamoto E, Seiki M (1994). A matrix metalloproteinase expressed on the surface of invasive tumor cells. Nature.

[B28] Gibbs DF, Warner RL, Weiss SJ, Johnson KJ, Varani J (1999). Characterization of matrix mtalloproteinses produced by rat alveolar macrophages. Am J Respir Cell Mol Biol.

[B29] Booke M, Bradford DW, Hinder F, Nishida K, Biondo NA, Traber LD, Traber DL (1997). Inhaled nitric oxide selectively reduces pulmonary hypertension after ovine smoke inhalation but does not improve oxygenation. J Burn Care Rehabil.

[B30] Marshall HE, Stamler JS (1999). Exhaled nitric oxide (NO), NO synthase activity, and regulation of nuclear factor (NF)-kappa B. Am J Respir Cell Mol Biol.

[B31] Sun SC, Elwood J, Beraud C, Greene WC (1994). Human T-cell leukemia virus type I Tax activation of NF-kappa B/Rel involves phosphorylation and degradation of I kappa B alpha and Rel A (p65)-mediated induction of the c-rel gene. Mol Cell Biol.

[B32] Deryckere F, Gannon F (1994). A one-hour minipreparation technique for extraction of DNA-binding proteins from animal tissues. Biotechniques.

[B33] Dahan S, Busuttil V, Imbert V, Peyron JF, Rampal P, Czerucka D (2002). Enterohemorrhagic Escherichia coli infection induces interleukin-8 production via activation of mitogen-activated protein kinases and the transcription factors NF-kappaB and AP-1 in T84 cells. Infect Immun.

[B34] Ishii M, Suzuki Y, Takeshita K, Miyao N, Kudo H, Hiraoka R, Nishio K, Sato N, Naoki K, Aoki T, Yamaguchi K (2004). Inhibition of c-Jun NH(2)-terminal kinase activity improves ischemia/reperfusion injury in rat lungs. J Immunol.

[B35] Morse D, Otterbein LE, Watkins S, Alber S, Zhou Z, Flavell RA, Davis RJ, Choi AMK (2003). Deficiency in the c-Jun NH_2_-terminal kinase signaling pathway confers susceptibility to hyperoxic lung injury in mice. AM J Physiol Lung Cell Mol Physiol.

[B36] Lai WC, Zhou M, Shankavaram U, Peng G, Wahl LM (2003). Differential regulation of lipopolysaccharide-induced monocyte matrix metalloproteinase (MMP)-1 and MMP-9 by p38 and extracellular signal-regulated kinase 1/2 mitogen-activated protein kinases. J Immunol.

[B37] Zhang X, Shan P, Sasidhar M, Chupp GL, Flavell RA, Choi AMK, Lee PJ (2003). Reactive oxygen species and extracellular signal-regulated kinase 1/2 mitogen-activated protein kinase mediate hyperoxia-induced cell death in lung epithelium. Am J Respir Cell Mol Biol.

[B38] Yue TL, Wang C, Gu JL, Ma XL, Kumar S, Lee JC, Feuerstein GZ, Thomas H, Maleeff B, Ohlstein EH (2000). Inhibition of extracellular signal-regulated kinase enhances ischemia/reoxygenation-induced apoptosis in cultured cardiac myocytes and exaggerates reperfusion injury in isolated perfused heart. Circ Res.

[B39] Ishizuka S, Yano T, Hagiwara K, Sone M, Nihei H, Ozasa H, Horikawa S (1999). Extracellular signal-regulated kinase mediates renal regeneration in rats with myoglobinuric acute renal injury. Biochem Biophys Res Commun.

[B40] Kang JL, Park W, Pack IS, Lee HS, Kim MJ, Lim CM, Koh Y (2002). Inhaled nitric oxide attenuates acute lung injury via inhibition of nuclear factor-κB and inflammation. J Appl Physiol.

[B41] Sacks M, Gordon J, Bylander J, Porter D, Shi XL, Castranova V, Kaczmarczyk W, Vandyke K, Reasor MJ (1998). Silica-induced pulmonary inflammation in rats: activation of NF-kappa B and its suppression by dexamethasone. Biochem Biophys Res Commun.

[B42] Schmitz ML, Stelzer G, Altmann H, Meisterernst M, Baeuerle PA (1995). Interaction of the COOH-terminal Transactivation Domain of p65 NF-κB with TATA-binding Protein, Transcription Factor IIB, and Coactivators. J Biol Chem.

[B43] Christman JW, Sadikot RT, Blackwell TS (2000). The role of nuclear factor-κB in pulmonary diseases. Chest.

[B44] Carter AB, Monick MM, Hunninghake GW (1999). Both Erk and p38 kinases are necessary for cytokine gene transcription. Am J Respir Cell Mol Biol.

[B45] Korus M, Mahon GM, Cheng L, Whitehead IP (2002). p38 MAPK-mediated activation of NF-kappaB by the RhoGEF domain of Bcr. Oncogene.

[B46] Leonardi A, Chariot A, Claudio E, Cunningham K, Siebenlist U (2000). CIKS, a connection to Ikappa B kinase and stress-activated protein kinase. Proc Natl Acad Sci U S A.

[B47] Funakoshi M, Tago K, Sonoda Y, Tominago S, Kasahara T (2001). A MEK inhibitor, PD98059 enhances IL-1-induced NF-κB activation by the enhanced and sustained degradation of IκB-α. Biochem Biophys Res Commun.

[B48] Briant L, Robert-Hermann V, Sivan V, Brunet A, Pouysségur, Devaux C (1998). Involvement of extracellular signal-regulated kinase module inHIV-mediatedCD4 signals controlling activation of nuclear factor-κB and AP-1 transcription factors. J Immunol.

[B49] Chen F, Demers LM, Vallyathan V, Ding M, Lu Y, Castranova V, Shi X (1999). Vanadate induction of NF-kappa B involves I kappa B kinase beta and SAPK/ERK kinase 1 in macrophages. J Biol Chem.

[B50] Dhawan P, Richmond A (2002). A novel NF-kappa B-inducing kinase-MAPK signaling pathway up-regulates NF-kappa B activity in melanoma cells. J Biol Chem.

[B51] Koch A, Giembycz M, Kazuhiro I, Lim S, Jazrawi E, Barnes PJ, Adcock I (2004). Mitogen-activated protein kinase modulation of nuclear factor-κB-induced granulocyte macrophage-colony-stimulating factor release from human alveolar macrophages. Am J Respir Cell Mol Biol.

